# Supplementary material for: Reorganization of pancreas circadian transcriptome with aging
Source: Aging (Albany NY). 2023 Aug 29;15(16):7909–21. doi: 10.18632/aging.204929 (PMC10497008; doi:10.18632/aging.204929)
Supplement: Supplementary Figures [file aging-15-204929-s001.pdf]

SUPPLEMENTARY FIGURES

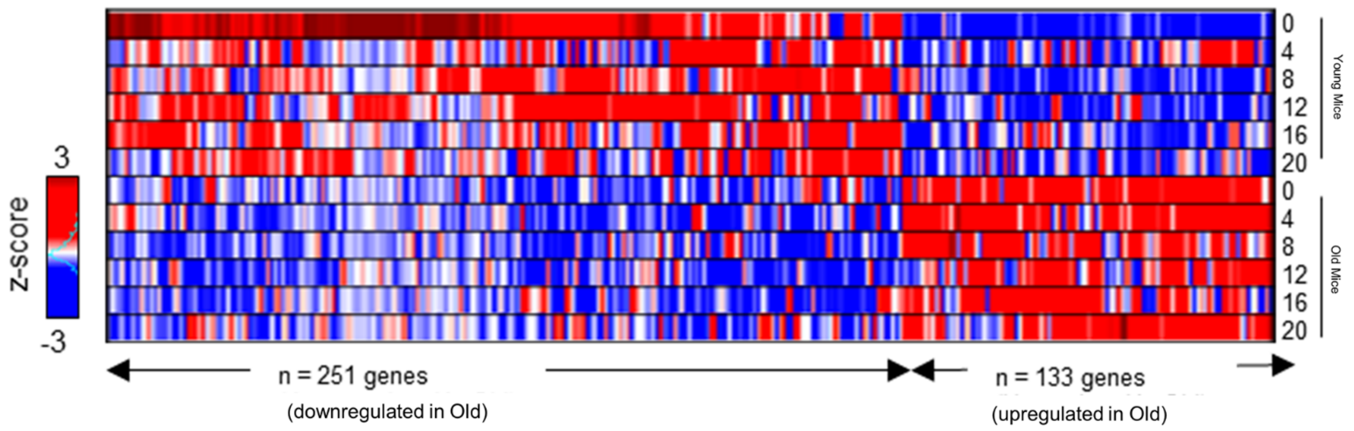

Supplementary Figure 1. Differentially expressed genes in mice pancreas. Heatmap of z-scored expression profiles for young and old RNA-Seq samples (n=12). Colors indicate relative (inter-sample) expression profiles for significantly up and down regulated genes (from panel 1A). Red: high TPM; Blue: Low TPM; White: Similar TPM. TPM = Transcripts per Million.

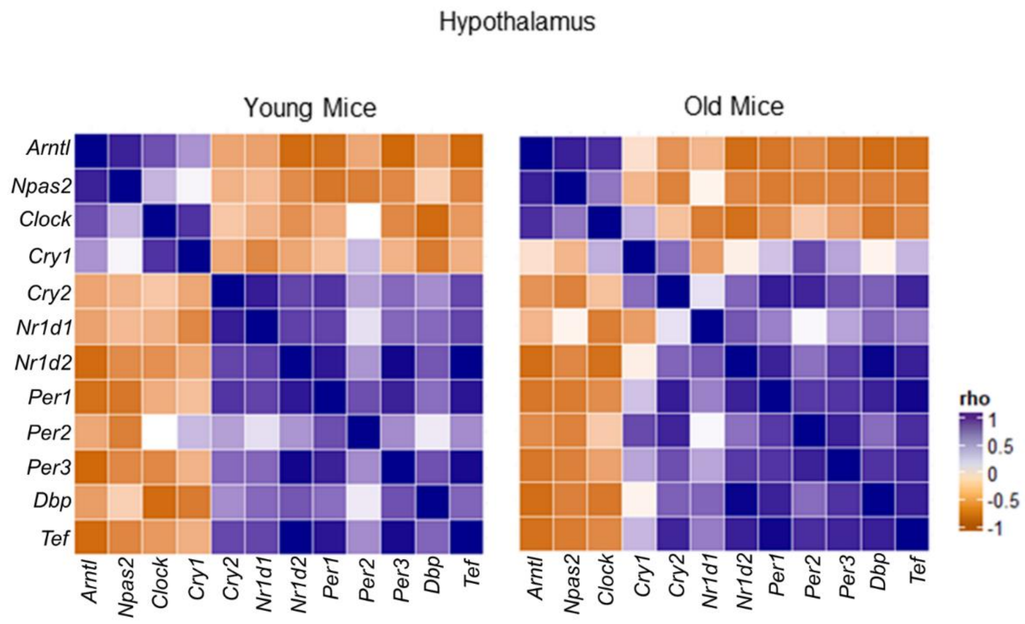

Supplementary Figure 2. Conserved central clock with aging. Clock correlation distance (CCD) of 12 core clock genes from young and older mice hypothalamus.

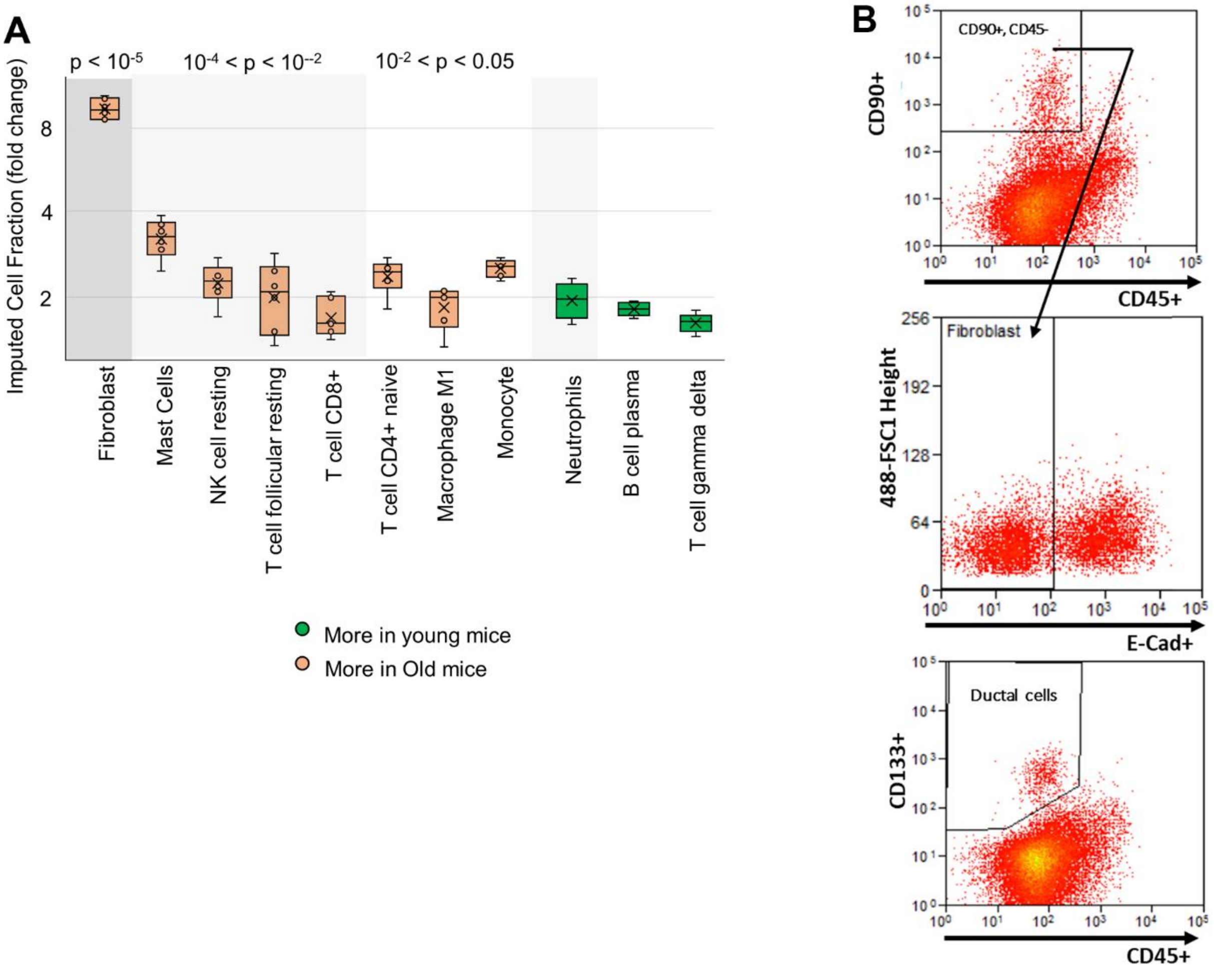

Supplementary Figure 3. Imputation of immune cell fractions in young and old pancreas. (**A**) Bar plots of the fold change for immune and stromal cell fractions obtained by imputation for young and old mice pancreas. p-value significances are color coded in grey. Cell types not significant are not shown. Green: More cells in young compared to old mice. Brown: More cells in old compared to young mice. (**B**) Representative flow cytometry workflow shows an example of gating scheme for fibroblasts in the pancreas tissue CD90+/CD45-/E-cadherin- (upper two panels) which was then normalized to the number of ductal epithelial cells; non-hematopoietic cells (CD45-) enriched in ductal (CD133+) markers (lowest panel) in each sample.

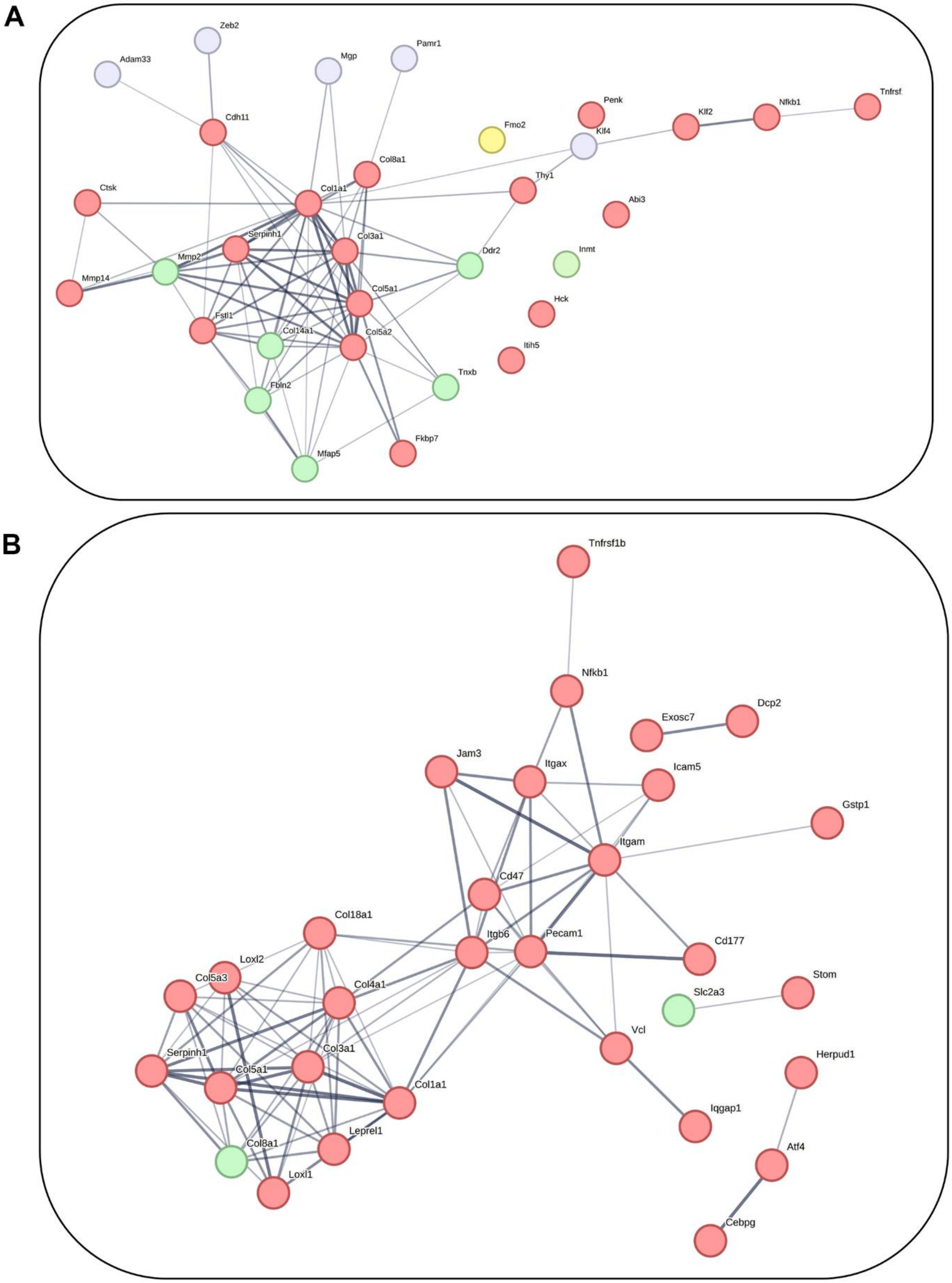

Supplementary Figure 4. Fibroblast activity is rhythmic in aged pancreas. (**A**) Hub proteins corresponding to genes that are markers for fibroblasts. Color: Red: Rhythmic in old pancreas. Green: Rhythmic in young pancreas. Grey: No change in rhythmicity. Yellow: Rhythmic in both young and old pancreas. Hub proteins were rhythmic in old pancreas at p< 0.0001. No significance was found for young pancreas. (**B**) Hub proteins corresponding to genes that are responsive to fibroblast signals. The color code is the same as panel (**A**). Hub proteins were rhythmic in old pancreas at p< 0.0001.
